# LncRNA SNHG14 regulates the DDP-resistance of non-small cell lung cancer cell through miR-133a/HOXB13 pathway

**DOI:** 10.1186/s12890-020-01276-7

**Published:** 2020-10-15

**Authors:** Li Xu, Yan Xu, Min Yang, Jia Li, Fang Xu, Bo-Lin Chen

**Affiliations:** 1 Thoracic Medicine Department 2, The Affiliated Cancer Hospital of Xiangya School of Medicine and Hunan Cancer Hospital, Central South University, No. 283, Tongzipo Road, Yuelu District, Changsha, 410013 Hunan Province PR China; 2 Respiratory Medicine Department 2, Hunan Children’s Hospital, Changsha, 410007 Hunan Province PR China

**Keywords:** NSCLC, LncRNA SNHG14, MiR-133a, HOXB13; DDP-resistance

## Abstract

**Background:**

Recently, long non-coding RNAs (lncRNAs) have been reported to be involved in regulating chemo-resistance of NSCLC, however, the role of lncRNA SNHG14 in the DDP-resistance of NSCLC remains unexplored.

**Methods:**

Relative expression of SNHG14, HOXB13 and miR-133a in DDP-resistant A549 (A549/DDP) cell and its parental cell A549 were measured using qRT-PCR. Cell proliferation viability of indicated A549/DDP cell was estimated via CCK-8 and colony formation experiments. Cell cycle and apoptosis were analyzed through flow cytometry. Expression of apoptosis-related protein and HOXB13 were detected via western blot. The interaction among SNHG14, HOXB13 and miR-133a was predicted by bioinformatics and validated by dual-luciferase reporter assay.

**Results:**

LncRNA SNHG14 and HOXB13 were upregulated while miR-133a was downregulated in A549/DDP cell line compared to A549 cell line. SNHG14 knockdown or miR-133a overexpression was demonstrated to increase the DDP-sensitivity of A549/DDP cells. SNHG14 was revealed to compete with HOXB13 for miR-133a binding in A549/DDP cells. Inhibition of miR-133a in A549 cells could reverse the promotive effects of SNHG14 knockdown on DDP-sensitivity, as well as the inhibitory effects on HOXB13 expression. HOXB13 overexpression was revealed to abolish the enhanced effects of miR-133a on the sensitivity of A549/DDP cell to DDP.

**Conclusion:**

Our findings demonstrated that SNHG14 was involved in the development of DDP-resistance of A549/DDP cells through miR-133a/HOXB13 axis, which may present a path to novel therapeutic stratagems for DDP resistance of NSCLC.

## Highlights

1. LncRNA SNHG14 knockdown and miR-133a overexpression increased the DDP-sensitivity of A549/DDP cell;
2. LncRNA SNHG14 targeted and inhibited miR-133a;
3. LncRNA SNHG14 regulates the DDP-resistance of non-small cell lung cancer cell through miR-133a/HOXB13 pathway

## Background

Lung cancer is one of the most common malignancies in the world that responsible for a considerable proportion cancer death [1]. The high mortality of lung cancer is largely due to the common diagnosis at advanced stages that impedes the curative treatment [2]. Without effective measures for early diagnosis where the tumors are still operable, and the lack of therapeutic options when the tumor is inoperable. These are the reasons why the five-year survival rate of NSCLC patients is less than 20% [3]. Cisplatin (DDP) is used as a first-line agent in the treatment of those postoperative or inoperable NSCLC patients [4]. The cancer-related death was previously demonstrated to decrease in the NSCLC patients who received DDP chemotherapy for 5 years compared to those untreated patients [5]. Nevertheless, individuals differently respond to DDP therapy and continuous DDP administration usually results in the occurrence of chemo-resistance, which frequently failed the clinical treatment [6]. Nowadays, DDP resistance is considered to be one of the most important impediments to the therapy of NSCLC patients.

MicroRNA (miRNA), featured by the short sequence (approximately 18–23 nucleotides), is a common subtype of endogenous non-coding RNA molecules [7]. The implication of miRNAs in modulating tumor-related gene expression have been well documented by numerous researches, showing it may function as a tumor repressor or an oncogene [8]. Accumulating data suggest that the tumor DDP-resistance could be modified by miRNAs [9], lighting up a novel research direction for tumor cell chemo-resistance. MiR-133a was revealed to be downregulated in NSCLC, and its expression level was negatively correlated with the lymphatic metastasis, tumor volume and clinical TNM stages [10]. In functional level, miR-133a was demonstrated to repress NSCLC cell proliferation and invasion in vitro [11, 12], however, up to now, there still has no study is performed to investigate whether miR-133a involves in the emergence of NSCLC chemo-resistance. Our preliminary bioinformatics experimental results predicted that HOXB13 could be targeted by miR-133a. Furthermore, HOXB13 is a specific transcription factor of prostate-lineage that predominately existed in the tail bud in the development of embryonic stage [13]. Recently, HOXB13 was reported to confer the DDP-resistance to lung cancer cells through networking with ABCG1/EZH2/Slug [14]. However, the molecular mechanisms of HOXB13 in DDP-resistance of NSCLC cells involved are still unclear.

As another important subtype of non-coding RNAs, long non-coding RNA (lncRNA) with more than 200 nucleotides has also revealed to affect the chemo-resistance of NSCLC through multiple mechanisms [15, 16]. Recently, lncRNA SNHG14 silencing was reported to repress NSCLC progression and enhance the NSCLC cell sensitivity to DDP [17]. According to bioinformatics prediction, lncRNA SNHG14 was a target of miR-133a.Therefore, we aimed to investigate whether lncRNA SNHG14 involves in the DDP-resistance of NSCLC cells by interacting with miR-133a/HOXB13 axis.

## Methods

### NSCLC cell lines and transfection

The parental NSCLC cell (A549) and DDP-resistant NSCLC cell (A549/DDP) were purchased from Cell bank of Chinese Academy of Sciences (Shanghai, China). Both A549 and A549/DDP cells were maintained in DMEM supplemented with fetal bovine serum (10%, Sigma, USA), penicillin (100 U/ml, Sigma) and streptomycin (100 mg/ml, Sigma) under 5% CO2 and 95% air. Thee specific sh-SNHG14, miR-133a mimics, miR-133a inhibitor, pcDNA3.1-HOXB13 (pc-HOXB13), and corresponding negative control (NC) were obtained from GenePharma (Shanghai, China). All these oligonucleotides were transfected into A549/DDP cells using Lipofectamine 3000 (Invitrogen, USA) following the instructions of manufacturers. After 48 h of transfection, cells were harvested for further research.

### Quantitative real-time PCR (qRT-PCR) analysis

TRIzol reagent (Invitrogen) was adopted to extract RNAs from indicated cells were prepared following the manufacturers’ protocol. The RNA quality was determined via a NanoDrop 2000 instrument (Thermo Scientific, USA), and 5 μg RNA was used as template to synthesis cDNA using a High-Capacity cDNA Reverse Transcription kit (Applied Biosystems, USA). SYBR Premix Ex Taq Kit (Takara, Tokyo) was adopted to conduct qRT-PCR on an ABI Prism 7700 system (PE Applied Biosystems, USA). Fold changes in RNA relative expression was counted through the 2^–ΔΔCt^ method. Primers were summarized as follows:

- SNHG14: forward, 5′-GGGTGTTTACGTAGACCAGAACC-3′; reverse, 5′-CTTCCAAAAGCCTTCTGCCTTAG-3′
- miR-133a: forward, 5′-CTGCAGCTGGAGAGTGTGCG-3′; reverse, 5′-GTGCTCTGGAGGCTAGAGGT-3′
- HOXB13: forward, 5′-ATGGAGCCCGGCAATTATGCCACC-3′; reverse, 5′-TTAAGGGGTAGCGCTGTTCTT-3′
- GAPDH: forward, 5′-GGCGTTCTCTTTGGAAAGGTGTTC-3′; reverse, 5′- GTACTCAGCGGCCAGCATCG -3′
- U6: forward 5′-CTC GCT TCG GCA GCA CA-3′, reverse 5′-AAC GCT TCA CGA ATT TGC GT-3′

### Cell proliferation viability analysis (CCK-8 and colony formation)

After 48 h of transfection, exponentially growing A549/DDP cells (1 × 10^5^ cells/well) were harvested and plated into 96-well plates supplemented with DDP (0, 2, 4, 6, AND 8 μg/ml), and then the proliferation viability of A549/DDP cells were tested by the Cell Counting Kit-8 (CCK-8; Dojindo Molecular Technologies, Japan) and colony formation assays after 24 h of DDP incubation. For CCK-8, the CCK-8 solution (200 μl) was added to the each well and then incubated at 37 °C for 2 h. The absorbance was measured by a microplate reader (Bio-Rad Laboratories, USA) at 450 nm. For colony formation assay, indicated A549/DDP cells (2000 cells/dish) were seeded into 35-mm culture dish and maintained at 37 °C. After 2 weeks, the visible colonies were fixed in 4% paraformaldehyde for 0.5 h, stained with 0.1% crystal violet solution for 10 min, and counted using a microscope.

### Cell cycle and apoptosis analysis

The A549/DDP cell cycle and apoptosis were estimated using flow cytometry. In brief, 48 h after transfection, cells were harvested for DDP treatment for additional 24 h. For the cell cycle assay, A549/DDP cells were fixed in 75% alcohol for 30 min, and then stained with propidium idoide (Sigma, MO, USA) for 15 min in dark. For cell apoptosis analysis, A549/DDP cells were washed with PBS followed by staining with Annexin V/PI kit (Vazyme, Nanjing, China) following the instructions of manufacturer. Finally, a BD Biosciences FACSCalibur Flow Cytometer (BD Biasciences, USA) was applied to detect the cell cycle and apoptosis.

### Dual-luciferase reporter assay

The wide type and mutant type miR-133a binding sequence of lncRNA SNHG14 and HOXB13 mRNA 3′-UTR were sub-cloned into the pmirGLO vector (Promega, Madison, USA) and named as SNHG14-WT, HOXB13-WT, SNHG14-MUT, and HOXB13-MUT, respectively. A549/DDP cells (1 × 10^6^ cells/wel) seeded in 96-well plates were co-transfected with constructed recombinant luciferase vectors and miR-133a mimics or mimics NC using Lipofectamine 3000 (Invitrogen). Luciferase intensity of A549/DDP cell was examined using the Dual-Luciferase Reporter Assay System (Promega) after 48 h of co-transfection.

### Western blot assay

Total proteins of indicated A549/DDP cells were isolated using the RIPA buffer (Beyotime, Beijing, China) contained protease inhibitors (Roche, Germany). Protein samples (50 μg) were isolated via 10% SDS-PAGE and then transferred into polyvinylidene difluoride (PVDF) membranes (Millipore, UK). After incubated in 5% skimmed milk for 2 h, the membranes were subjected for probe of primary antibodies that against Bcl-2 (1:2000, ab491583, Abcam), Bax (1:1000, ab199677, Abcam), cleaved-caspase-3 (1:1000, orb227889, Biorbyt) and HOXB13 (1:2000, ab53931, Abcam) overnight. Subsequently, the membranes were washed with PBS and then probed with corresponding secondary antibodies conjugated with horseradish peroxidase for 2 h. The signals were visible using an enhanced chemiluminescent reagent (ECL, Germany).

### Statistical analysis

All experiments were performed at least three times. Data were presented as mean ± standard deviation (SD), and all statistical analyses were conducted on SPSS (19.0 vision, IBM). Student’s t test or one-way analysis of variance followed by Tukey post hoc test was applied for the analysis of the difference between two or more groups. *P* value less than 0.05 was considered significant.

## Results

### LncRNA SNHG14 and HOXB13 were highly expressed, while miR-133a was lowly expressed in DDP-resistant NSCLC cell

**Fig. 1:**
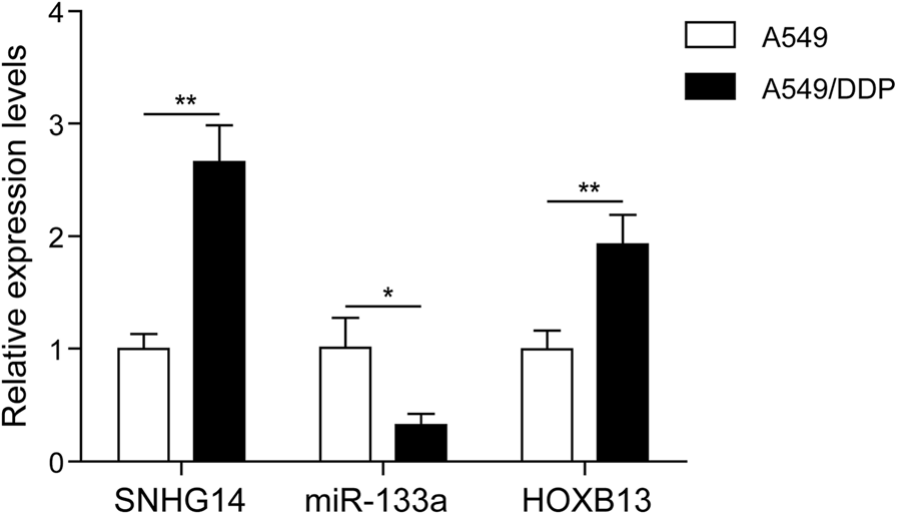
LncRNA SNHG14 and HOXB13 were highly expressed, while miR-133a was lowly expressed in DDP-resistant NSCLC cell. **a** Levels of lncRNA SNHG14, HOXB and miR-133a in normal A549 and DDP-resistant A549 (A549/DDP) cells were examined using qRT-PCR. *P < 0.05, **P < 0.01

To investigate whether lncRNA SNHG14, HOXB13 and miR-133a play roles in the chemo-resistance of NSCLC, we firstly examined their expression in parental NSCLC cell (A549) and DDP-resistant NSCLC cell (A549/DDP) using qRT-PCR. As results indicated that lncRNA SNHG14 and HOXB13 were remarkably increased, while miR-133a was significantly decreased in A549/DDP cells compared with A549 cells (Fig. 1a). The dysregulation of lncRNA SNHG14, HOXB13 and miR-133a in A549/DDP cells implied that they may be involved in the chemo-resistance of NSCLC.

### LncRNA SNHG14 silencing enhanced the sensitivity of A549/DDP cell to DDP

**Fig. 2:**
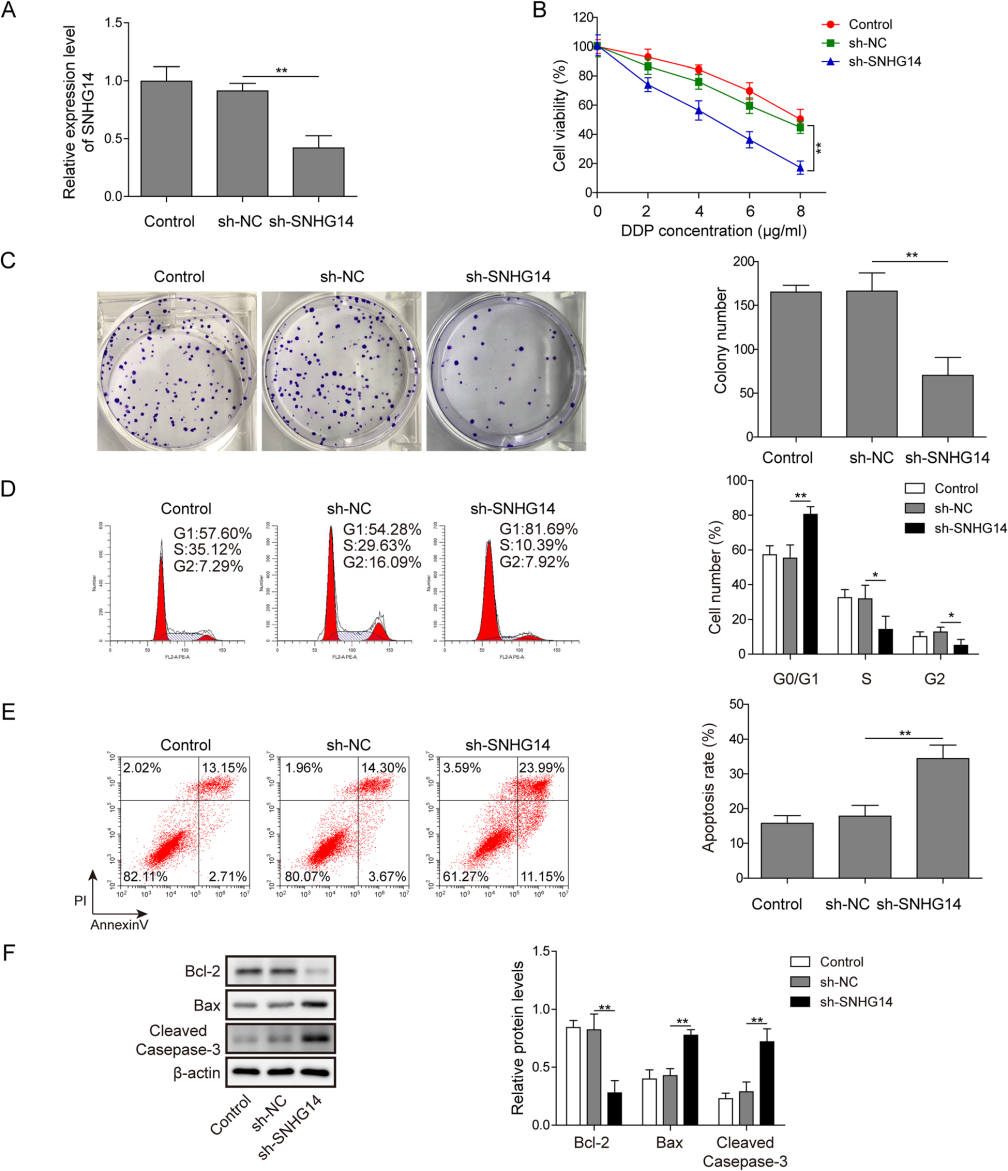
LncRNA SNHG14 silencing enhanced the sensitivity of A549/DDP cell to DDP. **a** A549/DDP cells transfected with sh-NC or sh-SNHG14. qRT-PCR examined expression of lncRNA SNHG14A549/DDP. **b** After transfected with sh-NC and sh-SNHG14, the cell viability of A549/DDP cell was detected via CCK-8 under the treatment of DDP with different concentration (0, 2, 4, 6 and 8 μg/ml). **c** The cell proliferation of sh-NC and sh-SNHG14 treated A549/DDP cell was detected by colony formation experiment in the presence of DDP (1 μg/ml). **d** and **e** After 48 h of sh-SNHG14 transfection, the A549/DDP cell was treated with DDP (1 μg/ml) and then subjected for cell cycle and apoptosis detection using flow cytometry analysis. **f** Protein expression levels of Bcl-2, Bax and cleaved-caspase-3 were measured by western blot in lncRNA SNHG14 blocked A549/DDP cells treated with DDP (1 μg/ml)

To further investigate the functional roles of lncRNA SNHG14 in the development of DDP resistance of A549/DDP cells, we silenced its expression in A549/DDP cells followed by the examination of cell viability, proliferation cycle and apoptosis. Results from qRT-PCR A549/DDP indicated a high knockdown efficiency of sh-SNHG14 in A549/DDP cells (Fig. 2a). The effects of A549/DDP cell viability under the treatment of DDP with different concentration (0, 2, 4, 6 and 8 μg/ml) were evaluated by CCK-8. The DDP-induced downregulation of A549/DDP cell viability was augmented in sh-SNHG14 group (Fig. 2b). Moreover, results from colony formation experiment demonstrated that lncRNA SNHG14 knockdown significantly reduced the A549/DDP cell number under the treatment of DDP (1 μg/ml) (Fig. 2c). In addition, flow cytometry was conducted in lncRNA SNHG14 silenced A549/DDP cells to estimate the influences of SNHG14 knockdown on A549/DDP cell cycle and apoptosis. In the presence of 1 μg/ml of DDP, lncRNA SNHG14 silencing resulted in a significant upregulation of cell number in G0/G1 phases and a remarkable downregulation of S phase (Fig. 2d). The A549/DDP cell apoptosis rate was significantly increased in sh-SNHG14 transfected group (Fig. 2e). In the western blot detection of apoptosis-related protein expression, we found that sh-SNHG14 transfection significantly decreased Bcl-2 expression, while increased Bax and cleaved-caspase-3 expression (Fig. 2f). Taken together, these findings strongly supported that lncRNA SNHG14 knockdown enhanced the sensitivity of A549/DDP cells to DDP.

### miR-133a treatment enhanced the sensitivity of A549/DDP cell to DDP

**Fig. 3:**
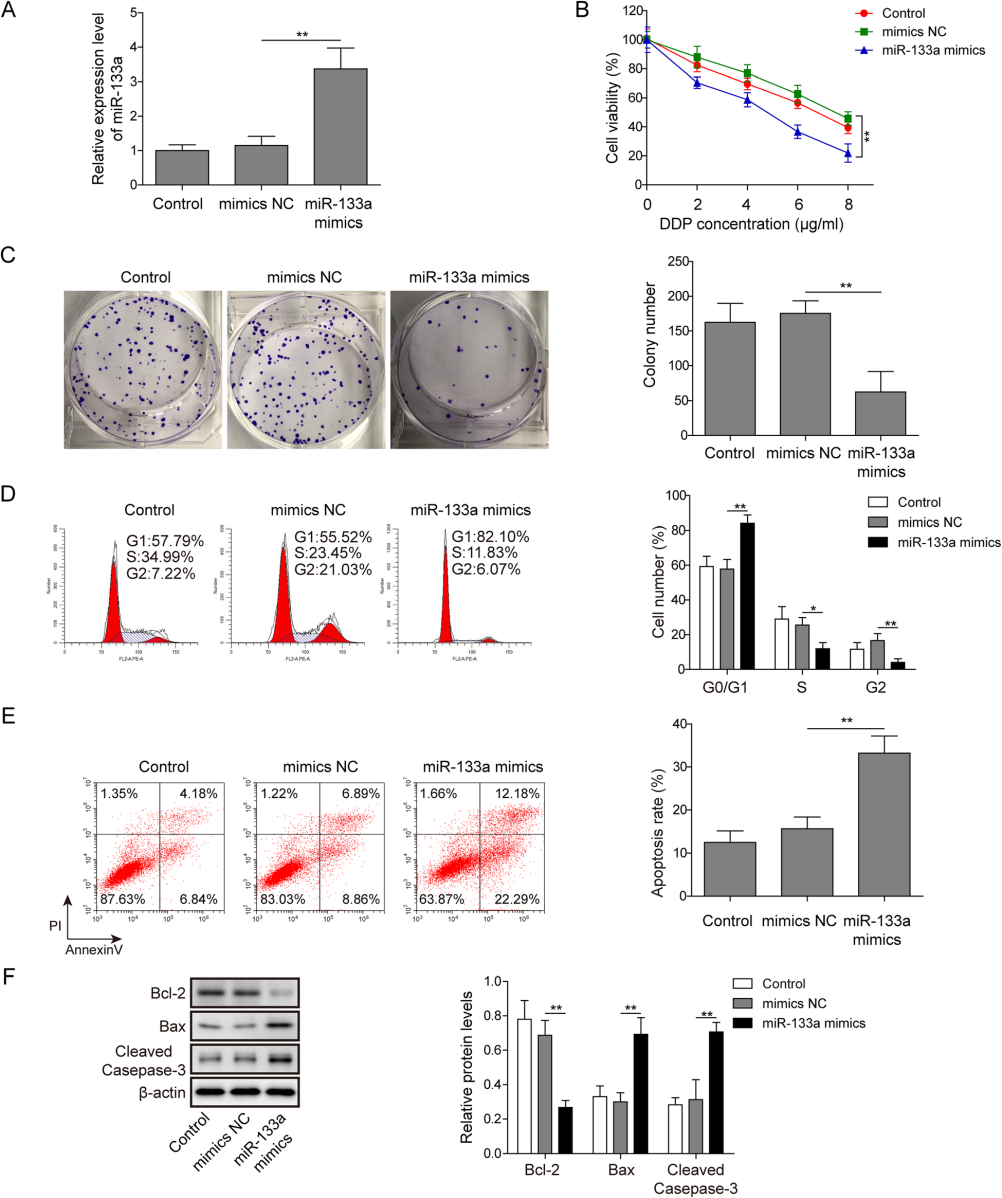
miR-133a treatment enhanced the sensitivity of A549/DDP cell to DDP. **a** qRT-PCR was conducted in mimics NC and miR-133a mimics transfected A549/DDP cells to examine the overexpression efficiency of miR-133a. **b** After transfected with mimics NC and miR-133a mimics for 48 h, the cell viability of A549/DDP cell was examined in the presence of DDP (1 μg/ml) by CCK-8. **c** The cell proliferation of mimics NC and miR-133a mimics treated A549/DDP cell was detected by colony formation experiment in the presence of DDP (1 μg/ml). **d** and **e** After 48 h of mimics NC and miR-133a mimics transfection, the A549/DDP cell was treated with DDP (1 μg/ml) and then subjected for cell cycle and apoptosis detection using flow cytometry analysis. **f** Protein expression levels of Bcl-2, Bax and cleaved-caspase-3 were measured by western blot in miR-133a mimics transfected A549/DDP cells treated with DDP (1 μg/ml)

We then explored the functional roles of miR-133a in the development of DDP resistance of NSCLC by overexpressing its expression in A549/DDP cells, and then estimated the cell viability, proliferation, cycle and apoptosis of miR-133a overexpressed A549/DDP cells. The overexpression efficiency of miR-133a in A549/DDP cells was determined by qRT-PCR (Fig. 3a). CCK-8 was utilized to detect the influences of miR-133a overexpression on A549/DDP cell viability in the presence of different concentrations of DDP (0, 2, 4, 6 and 8 μg/ml). As results showed that the A549/DDP cell viability of miR-133a mimics group was significantly lower than that of sh-NC group (Fig. 3b). Forthermore, miR-133a overexpression led to a significant reduction of A549/DDP cell number (Fig. 3c). In addition, the influences of miR-133a mimics treatment on A549/DDP cell cycle and apoptosis in the presence of 1 μg/ml of DDP were estimated via flow cytometry. As results demonstrated that miR-133a overexpression remarkably increased the A549/DDP cell number of G0/G1 phases, while reduced the A549/DDP cell number of S phase (Fig. 3d). Moreover, miR-133a overexpression led to a significant upregulation of A549/DDP cell apoptosis rate induced by DDP (Fig. 3e). Similar to lncRNA SNHG14 knockdown, we also found that miR-133a overexpression significantly downregulated the protein expression of Bcl-2 while upregulated the protein expression of Bax and cleaved-caspase-3 in A549/DDP cells treated with DDP (Fig. 3f). These data highly suggested that miR-133a overexpression increased the DDP sensitivity of A549/DDP cells.

### LncRNA SNHG14 competed with HOXB13 for miR-133a binding

**Fig. 4:**
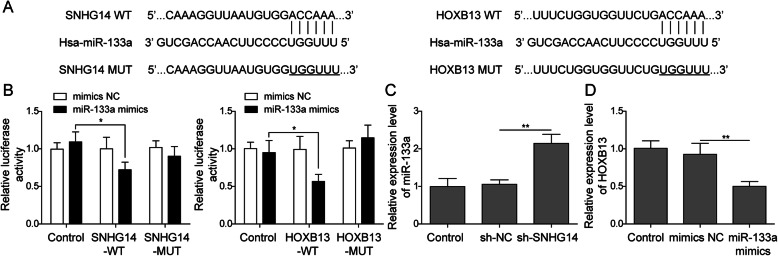
LncRNA SNHG14 competed with HOXB13 for miR-133a binding. **a** Putative miR-133a binding sequence of lncRNA SNHG14 and HOXB13. **b** Dual-luciferase reporter experiment was conducted in A549/DDP cell to validate the interplay between SNHG14 and miR-133a, as well as miR-133a and HOXB13. **c** miR-133a level was examined in sh-NC and sh-SNHG14 transfected A549/DDP cell by qRT-PCR. **d** qRT-PCR analysis of HOXB13 in mimics NC and miR-133a mimics transfected A549/DDP cell

Numerous studies have shown that lncRNAs participated almost in every aspect of the biological processes of tumor progression by competitively binding to specific miRNAs and thus releasing target genes. Therefore, bioinformatics analysis was performed to predict the target miRNAs which could interact with lncRNA SNHG14. We found that lncRNA SNHG14 possessed complementary sequence of miR-133a. Moreover, HOXB13 was also found to possess the complementary sequence of miR-133a (Fig. 4a). To validate the direct binding between lncRNA SNHG14 and miR-133a, as well as miR-133a and HOXB13, dual-luciferase reporter assay was conducted in A549/DDP cells. Results demonstrated that co-transfection with SNHG14-WT or HOXB13-WT and miR-133a mimics caused a significant repression of luciferase activity, while co-transfection with SNHG14-MUT or HOXB13-MUT and miR-133a mimics exhibited no obvious effects on the luciferase activity (Fig. 4b). Besides, we found thatA549/DDP lncRNA SNHG14 knockdown resulted in an upregulation of miR-133a in A549/DDP cells (Fig. 4c), and miR-133a overexpression caused a downregulation of HOXB13 (Fig. 4d). Together, these results indicated that lncRNA SNHG14 competed with HOXB13 for miR-133a binding.

### HOXB13 overexpression reversed the enhanced effects of miR-133a on the sensitivity of A549/DDP cell to DDP

**Fig. 5:**
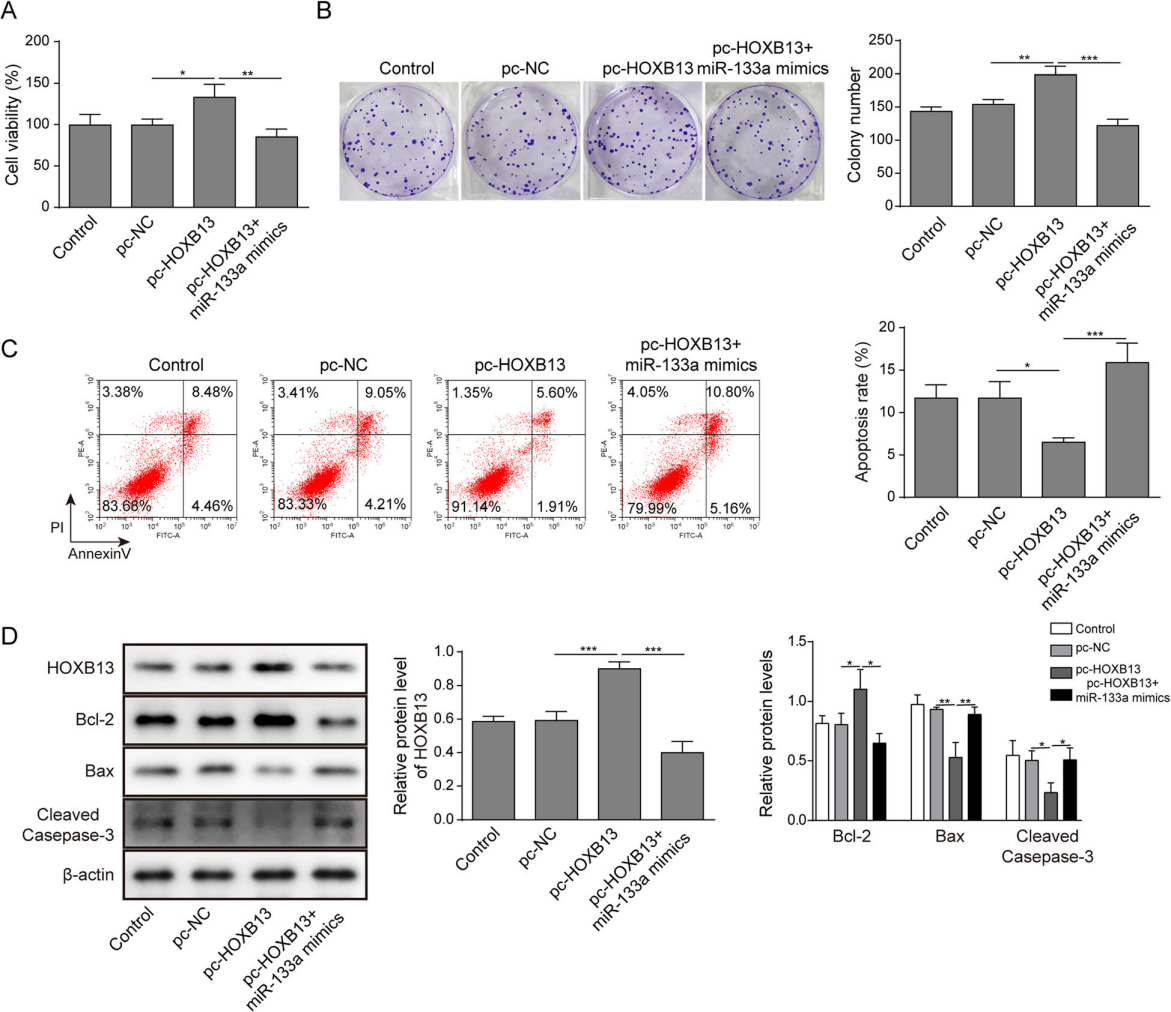
HOXB13 overexpression reversed the enhanced effects of miR-133a on the sensitivity of A549/DDP cell to DDP. A549/DDP cells under DDP treatment (1 μg/ml) were co-transfected with pc-HOXB13 and miR-133a mimics, then the cell viability, proliferation, and apoptosis was determined by **a** CCK-8, **b** colony formation and **c** flow cytometry analysis, respectively. **d** The protein expression of HOXB13, Bax, Bcl-2 and caspase-3 in pc-HOXB13 and miR-133a mimics co-transfected A549/DDP cells were assessed by western blot

To investigate whether HOXB13 plays a role in the enhanced effects of miR-133a on the sensitivity of A549/DDP cell to DDP, we treated A549/DDP cells with pc-HOXB13 and miR-133a mimics followed by the examination of cell viability, proliferation and apoptosis in the presence of DDP. The results indicated that overexpression of HOXB13 dramatically increased A549/DDP cell viability and colony-formation ability. However, co-transfection of pc-HOXB13 and miR-133a mimics reversed the upregulation of cell viability and colony-formation ability induced by pc-HOXB13 transfection in A549/DDP cells (Fig. 5a-b). Moreover, by using flow cytometry analysis, we revealed that pc-HOXB13 transfection sharply reduced A549/DDP cell apoptosis, while co-transfection of pc-HOXB13 and miR-133a mimics abolished the reduction of A549/DDP cell apoptosis induced by pc-HOXB13 transfection (Fig. 5c). Additionally, results from western blot indicated that overexpression of HOXB13 dramatically increased the expression of HOXB13 and Bcl-2 while decreased the expression of Bax and cleaved caspase-3 in the A549/DDP cells, however, co-transfection of pc-HOXB13 and miR-133a mimics abolished the dysregulation of these molecules (Fig. 5d). These findings suggested that overexpression of HOXB13 repressed the sensitivity of A549/DDP cell to DDP through antagonizing miR-133a.

### miR-133a/HOXB13 pathway involved in the lncRNA SNHG14 regulation of A549/DDP cell sensitivity to DDP

**Fig. 6:**
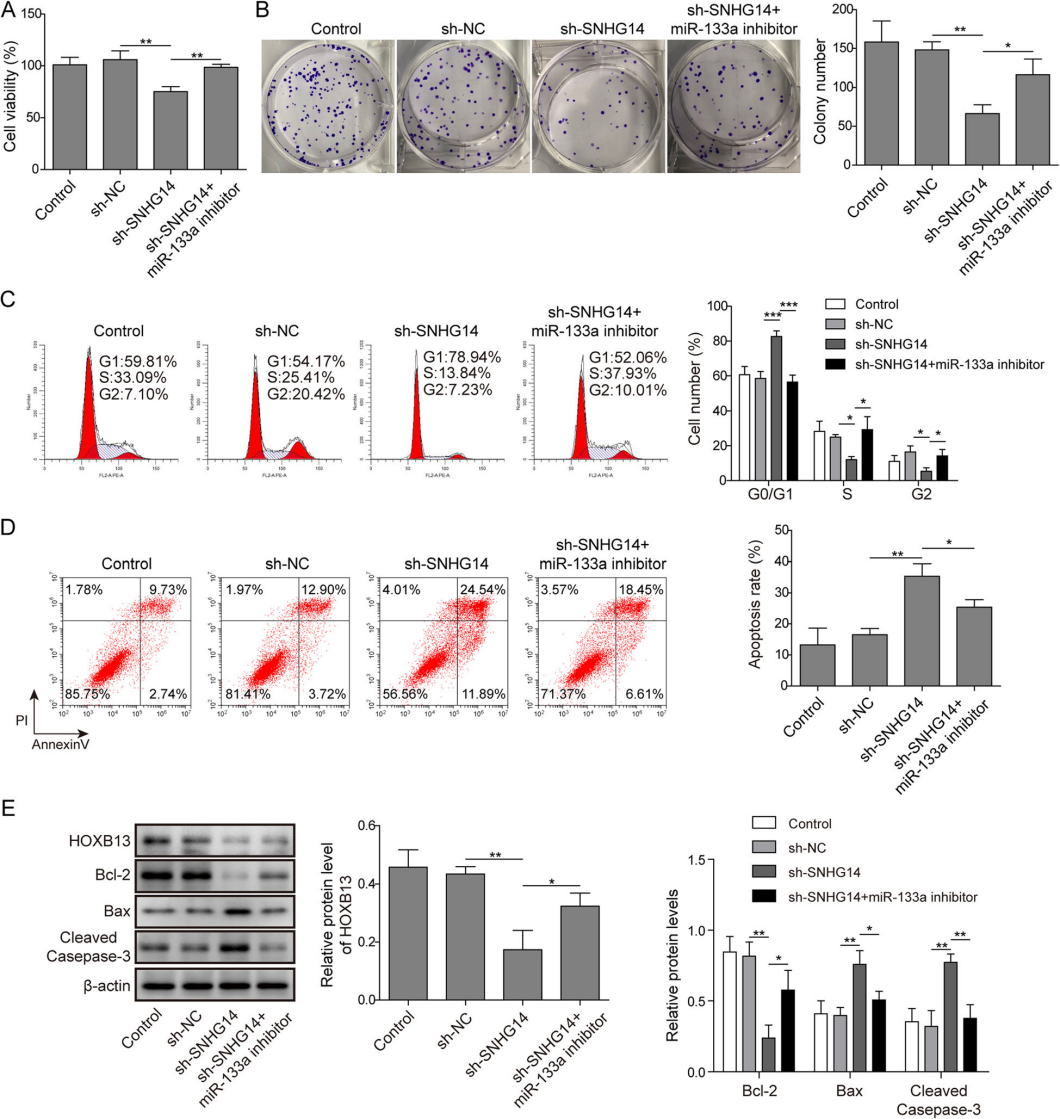
miR-133a/HOXB13 pathway involved in the lncRNA SNHG14 regulation of A549/DDP cell sensitivity to DDP. **a** and **b** A549/DDP cells under DDP treatment (1 μg/ml) were co-transfected with sh-SNHG14 and miR-133a inhibitor, then the cell viability and proliferation was determined by CCK-8 and colony formation experiments. **c** and **d** Flow cytometry was adopted to analyze the effects of co-transfection of sh-SNHG14 and miR-133a inhibitor on the cell cycle and apoptosis of A549/DDP cell in the presence of DDP (1 μg/ml). **e** Western blot was utilized to test the protein levels of Bcl-2, Bax, cleaved-caspase-3 and HOXB13 in A549/DDP cells co-transfected with sh-SNHG14 and miR-133a inhibitor under DDP treatment (1 μg/ml)

Considering the interaction among lncRNA SNHG14, HOXB13 and miR-133a in A549/DDP cells, we further investigated whether miR-133a is involved in the process of lncRNA SNHG14 regulating the DDP-resistance of NSCLC through HOXB13 pathway. We have shown that sh-SNHG14 transfection could significantly decrease the A549/DDP cell viability in the presence of DDP while this repression of cell viability caused by sh-SNHG14 treatment was reversed by the miR-133a inhibitor (Fig. 6a). Similarly, in colony formation assay, the sh-SNHG14 treatment induced reduction of A549/DDP cell number was recovered by the co-transfection of sh-SNHG14 and miR-133a inhibitor (Fig. 6b). Moreover, the sh-SNHG14 treatment induced upregulation of G0/G1 phases cell number and downregulation of S phase cell number were counteracted partially by inhibition of miR-133a (Fig. 6c). The co-transfection of sh-SNHG14 and miR-133a inhibitor also abolished the promotive effects of sh-SNHG14 transfection on A549/DDP cell DDP-inducted apoptosis (Fig. 6d). In the western blot assay, we demonstrated that co-transfection of sh-SNHG14 and miR-133a inhibitor could reverse the sh-SNHG14 transfection induced Bcl-2 downregulation and Bax, cleaved-caspas-3 upregulation (Fig. 6e). In addition, we found that lncRNA SNHG14 knockdown significantly reduced the epression of HOXB13, however, co-transfection of sh-SNHG14 and miR-133a inhibitor abolished this effect (Fig. 6e). These results implied that miR-133a/HOXB13 pathway involved in the lncRNA SNHG14 regulation of A549/DDP cell sensitivity to DDP.

## Discussion

Although the exact mechanisms underlying DDP-resistance remain largely unclear, it is certain that this problem could not be conquered through targeting any single mechanism strategy [18, 19]. Galuzzi et al. have proposed four distinct DDP-resistance mechanisms: (i), by reducing the cellular concentration of DDP to prevent its binding with DNA; (ii), repairing the DDP-DNA adducts; (iii), restoring the dysregulated signaling pathways in response to DNA damage caused by DDP; (iiii), through indirect mechanisms that do not involve DDP-related signals but confer resistance to DDP-induced death [20]. Considering the substantial genes, proteins and signal cascades involved in the emergence of DDP-resistance [21], it is likely to fail if we focus on any single mechanism-targeted strategy. Here, we demonstrated that lncRNA SNHG14, through miR-133a/HOXB13 pathway, played as a regulator during the development of DDP-resistance of NSCLC cells, providing a novel signal cascade that may be used as a promising therapeutic target for the overcome of DDP-resistance of NSCLC.

With the advancement of technologies in DNA sequencing and bioinformatics analysis, numerous of lncRNAs and miRNAs have been proven to be involved in the drug-resistance of NSCLC through multiple mechanisms [16, 22]. For example, Ma LY et al. have shown the repressive effects of lncRNA TRPM2-AS knockdown on NSCLC cell DDP-resistance, moreover, they also demonstrated this effects was p53- p66shc pathway dependent [23]. Moreover, miR-488 was revealed to repress NSCLC cell proliferation and reduce NSCLC cell sensitivity to DDP via the activation of eIF3a-mediated NER signaling cascade [24]. LncRNA SNHG14 participates in the progression of various human tumors, such as glioma, cervical cancer and NSCLC [25–27]. Recently, it was also involved in the drug-resistance of tumor cells. Dong H et al. reported that SNHG14 enhanced the trastuzumab resistance of breast cancer cells by modulating PABPC1 level trough H3K27 acetylation [28]. Moreover, SNHG14 was proved to confer gefitinib resistance in NSCLC cell by increasing ABCB1 level through sponging miR-206-3p [29]. In line with previous researches, our study found a significant upregulation of lncRNA SNHG14 in A549/DDP cell, and SNHG14 knockdown resulted in an enhancement of DDP-sensitivity of A549/DDP cell.

It was reported that miR-133a could increase the DDP-sensitivity of Hep-2 and vincristine resistant Hep-2v cell through reducing ATP7B level [30]. Although miR-133a plays a role in the multiple biological processes of NSCLC cell, including proliferation, migration and invasion [11, 12], its implication in NSCLC DDP-resistance remains no report. In this study, we found miR-133a was downregulated in DDP-resistant A549/DDP cells, and its overexpression increased the DDP-sensitivity of A549/DDP cells. Taken together, lncRNA SNHG14 and miR-133a were involved in the DDP-resistance of NSCLC.

The involvement of lncRNAs in tumor cell drug-resistance is largely mediated by miRNAs and thus affected their downstream target genes [31]. For instance, LncRNA SNHG14 was demonstrated to increase gemcitabine resistance of pancreatic cancer cells by interacting with miR-101 [32]. In the present study, lncRNA SNHG14 was firstly found to compete with HOXB13 for miR-133a binding. Functionally, miR-133a inhibition abolished the repressive effects of sh-SNHG14 on DDP-resistance and HOXB13 expression. Moreover, HOXB13 overexpression reversed the enhanced effects of miR-133a on the sensitivity of A549/DDP cell to DDP.

## Conclusion

In conclusion, our findings provided evidences that lncRNA SNHG14 regulated the DDP-resistance of NSCLC cell in vitro by increasing HOXB13 expression through miR-133a. Thus, lncRNA SNHG14/miR-133a/HOXB13 regulatory network might be promising therapeutic target for NSCLC drug-resistance.

## Data Availability

All data generated or analyzed during this study are included in this published article.

## Abbreviations

LncRNAs: Long non-coding RNAs
NSCLC: Non-small cell lung cancer
SCLC: Small-cell lung cancer
MicroRNAs: MiRNAs
Lnc RNA SNHG14: LncRNA-small nucleolar RNA host gene 14
HOXB13: Homeobox B13
